# The art of equity: critical health humanities in practice

**DOI:** 10.1186/s13010-023-00149-1

**Published:** 2023-12-13

**Authors:** Irène P. Mathieu, Benjamin J. Martin

**Affiliations:** 1 Department of Pediatrics; Program in Health Humanities, Center for Health Humanities & Ethics, University of Virginia School of Medicine, PO Box 800386, Charlottesville, VA 22908 USA; 2 Department of Internal Medicine; Program in Health Humanities, Center for Health Humanities & Ethics, University of Virginia School of Medicine, PO Box 800386, Charlottesville, VA 22908 USA

**Keywords:** Health humanities, Narrative medicine, Health equity, Social determinants of health, Structural competency, Medical education

## Abstract

**Background:**

The American Association of Medical Colleges has called for incorporation of the health humanities into medical education, and many medical schools now offer formal programs or content in this field. However, there is growing recognition among educators that we must expand beyond empathy and wellness and apply the health humanities to questions of social justice – that is, critical health humanities. In this paper we demonstrate how this burgeoning field offers us tools for integrating social justice into medical education, utilizing the frameworks of critical consciousness and structural competency.

**Practice of health humanities:**

Critical health humanities can be applied at multiple levels of learners, and in a variety of contexts. We are two physician-writers who have developed several educational programs that demonstrate this. We taught a seminar that introduced first-year and second-year undergraduates to concepts such as social determinants of health, intergenerational trauma, intersectionality, resilience, and cross-cultural care through works of fiction, poetry, film, podcasts, stand-up comedy, and more. Through creative projects and empathic reflection, students engaged with the complexities of structural forces that create and maintain health disparities.

Medical students in their clinical years can engage in critical health humanities learning experiences as well. We teach several multidisciplinary electives that address social (in)justice in medicine, as well as mentor fourth-year students engaged in independent electives that foster critical awareness around health equity and ethics.

Beyond the classroom, we have actively engaged in critical health humanities practices through story slams, literary journal clubs, conference presentations, and Grand Rounds. Through these activities we have included learners at GME and CME levels. These examples also demonstrate how community engagement and multidisciplinary partnerships can contribute to the practice of critical health humanities.

**Conclusion:**

In this paper, we explore the growing field of critical health humanities and its potential for teaching health equity through narrative practices. We provide concrete examples of educational activities that incorporate critical consciousness and structural competency – frameworks we have found useful for conceptualizing critical health humanities as a pedagogical practice. We also discuss the strengths and challenges of this work and suggest future directions.

## Background

Over the last decade, evidence of the value of the health humanities has accumulated, and the American Association of Medical Colleges has called for its inclusion in medical education [1]. The majority of medical schools now offer formal health humanities programs or content in their curricula [2]. Practitioners of the health humanities also have begun to call for recognition of the larger role this field might play in educational work that “moves beyond empathy and wellness to prioritize the intersection between humanities scholarship and social justice” [3]. The health humanities can do this through a variety of mechanisms, including by disrupting automatic beliefs and perceptions, cultivating learners’ tolerance for ambiguity, introducing students and trainees to a multiplicity of voices, and illuminating the historical and social forces that underpin health outcomes [4]. The critical health humanities represents an opportunity to enmesh study of the human body and its pathologies with the social, political, historical, and environmental contexts in which that body exists, so that biomedical and humanities disciplines are “productively entangled” to engender more nuanced and holistic perspectives among learners [5]. In this commentary, we will describe innovative approaches to operationalizing this emerging role of the humanities in medical education.

While there are multiple frameworks for teaching socio-cultural contextual awareness, we have found structural competency and critical consciousness to be particularly useful for conceptualizing the pedagogy of critical health humanities. Structural competency is a medical education paradigm that calls for training on how socially constructed variables - such as race, immigration status, socio-economic status, incarceration history, and more - impact the ways in which people experience health, illness, and health care [6]. It complements cultural humility, which is another important framework for understanding the role of the arts and humanities in revealing to learners glimpses of the vast diversity of human experience. The term “critical consciousness,” or conscientização, first coined by Paulo Freire, refers to awareness of one’s own social position and ability to transform society, and has been invoked as a key objective of medical education [4]. Taken together, we posit that the critical health humanities offers a means for developing students’ structural competency through the narrative practices of close reading and reflective writing [7]. Close reading of literary texts can train (future) clinicians to “closely read” their patients’ social and political contexts, and can also generate what we term “structural compassion” in ways that quantitative pedagogy on health disparities alone cannot. In practice, an example of “structural compassion” might be a clinician’s consideration of social, political, and economic factors that might impact a patient’s ability to follow a treatment plan and open-ended questions to better understand the role of these factors in the patient’s life, rather than incuriously labeling such a patient as “noncompliant.”

## The practice of critical medical humanities

How might the critical health humanities be taught? Our work at the University of Virginia offers a window into the diverse ways learners at different levels can benefit from this field. We are physician-writers who have published both scholarly and creative works relevant to the health humanities. We are core faculty in our university’s Center for Health Humanities and Ethics (CHHE), which serves as the hub for educational offerings related to the health humanities. We have focused our pedagogy on the critical health humanities, and in this paper we offer several concrete examples of this work for learners at multiple levels.

We designed a seminar for first-and second-year undergraduates at our institution called “Bodies & Books: Literature of Health (In)equity”, which explored social determinants of health (SDoH) through the analysis of art and literature. These “first-year seminars” are designed to facilitate innovative and interdisciplinary educational opportunities for small groups of undergraduate students in their first or second year of college. Our seminar included five self-selected students with a range of interests, from pre-nursing to international development. Concepts like biopsychosocial models of care, intergenerational trauma, intersectionality, resilience, and cross-cultural care were brought to life by characters and situations depicted in the selected works, which included fiction, creative nonfiction, poetry, film, podcast, stand-up comedy, and more. We also assigned creative projects aimed at inculcating the practice of empathic reflection as an active and generative process integral to redressing structural forces that exacerbate SDoH. For undergraduates interested in public health, translational research, or clinical care, this course introduced core concepts of SDoH via media that are fundamentally rooted in imaginative empathy, establishing as normative the notion that patients are complex individuals in constant dynamic engagement with greater sociopolitical and environmental forces.

One specific example is our use of poet Bettina Judd’s work on the intersection of gender and race in her experiences as a patient, imagined as part of an historical continuum of health disparities that she traces to Anarcha, Betsey, and Lucy, the enslaved women who were unwilling participants in J. Marion Sims’s gynecological research in her stirring book *Patient*. Students were challenged to consider the emotional register of Judd’s poems, the poetic devices she wields to illustrate the impact of history on the present, and what her work says about inequities in our health care system. Four of the five students who completed the “Bodies & Books” seminar responded to a survey we created to evaluate the course. They universally expressed that the course deepened both their understandings of SDoH and their critical literary analysis skills. They reported enjoying the course, with the main critique being the all-virtual format (per university stipulations at that point during the COVID-19 pandemic). Several students also reported that the course influenced their career goals.

For medical students in their active clinical years, critical health humanities also affords a comprehensive understanding of the system in which they play active roles. We have infused two decades-long CHHE courses in the health humanities – Literature and Medicine and Calls of Medicine – with critical consciousness and structural competency learning objectives and curricula elements. These courses are taught in the spring semester of the fourth year of medical school, which positions them as capstone courses during which students integrate clinical experiences with their identities, values, and goals as emerging physicians. Typical enrollment for these heavily discussion-based, seminar-style courses is 7–12 students, and includes a mixture of medical students in the Hook Scholars program (a selective health humanities curricular track) and others, consistently including some students with little or no background or training in the arts or humanities.

Literature and Medicine is a two-week elective for medical students rooted in the dual practices of seminar-style discussion of pre-assigned literary texts and creative writing. Each class focuses on a specific overarching concept with attention to social and structural determinants of health woven throughout. Before each session, students read, watch, or listen to pre-assigned works drawn from diverse genres, including but not limited to poetry, fiction, creative non-fiction, podcast, film, and more. In class, we pose contemplative questions that form the basis for robust discussions of each piece, with equal focus on content and craft. With a special emphasis on the rhetorical opportunities inherent to fiction and poetry, we assign writing prompts both in class and as homework, designed to elicit salient insights around their patients’ – and their own – identities and positionalities in the clinical world and beyond.

For instance, we teach Carmen Maria Machado’s short story “Eight Bites,” a gothic, magical realism tale that explores the psychic toll of bariatric surgery – and, more broadly, the trauma of gendered expectations around body size. After discussing the story’s literary devices that complicate our medical understanding of obesity and weight loss, students embark on a creative writing exercise in which they envision how the main character in “Eight Bites” might experience a clinic visit with her health care provider. It is an exercise in clinical humility, as students consider the impacts of social and familial relationships, societal expectations and norms, and a hypothetical patient’s interior life and emotions on what they have been taught to view as a primarily physiological problem.

Calls of Medicine is a multidisciplinary elective that explores the meaning of medicine as a vocation. Through seminar discussions of art and critical texts – often involving the authors of the works being discussed – students collaborate on addressing this overarching question: what are the ethical obligations a physician has to society, and how are we called on in practical terms to address social inequities? In Calls of Medicine we are joined in person by poet Brian Teare, a faculty member in the Department of English, who discusses his seminal book on experiencing the failures of the medical system as a patient with chronic illness. The poems and ensuing discussion challenge students to rethink their assumptions about how disease may or may not adhere to the narrative logics of the structured clinical cases that have formed the basis of their education thus far. Teare’s work illustrates how physicians are called upon to continuously interrogate our assumptions about how diseases behave in the lives of our patients and how such assumptions can compromise the therapeutic relationship, and to understand the limits and potential of differential expertise held by patients and health care providers. This inter-departmental collaboration also exemplifies the role of relationships with scholars of the humanities and arts in creating robust critical health humanities content. In addition to Teare’s regularly guest-teaching in both Literature & Medicine and Calls of Medicine, the authors have collaborated with the poet in public readings and discussions of his work, and he has shared first author IM's poetry with his creative writing students, particularly those with an interest in medicine or the health sciences. All three of us have benefited intellectually and psychologically from this cross-disciplinary relationship.

Lastly, independent creative projects are available to fourth-year students in either two- or four-week rotations. Through interactive and intensive projects closely supervised by faculty, medical students revisit clinical or personal experiences with the goal of either clarifying or complicating key themes in the relationships explored. Student projects have ranged from original works of creative writing exploring illness identities to research-driven projects fusing topics in clinical medicine and music or visual art.

We have also led or participated in meaningful critical health humanities work outside the classroom. In recent years there has been a proliferation of story sharing opportunities in medical education spaces. These include national health humanities conferences; story slams; journal clubs that center literary works; formal opportunities for patients to share their stories during conferences or Grand Rounds; and the invitation of “professional” storytellers to such educational events. One such instance occurred at our institution in 2019. Led by IM, the Department of Pediatrics invited nationally renowned poet Javier Zamora to speak at Grand Rounds and Medical Center Hour (a lecture series that is a program of the CHHE) about his work related to his experience crossing the U.S.-Mexico border as an unaccompanied minor when he was nine years old. In addition, the Department partnered with a local extramural organization, of which IM was then a board member, to create a community-facing event in which Zamora gave a reading and led a poetry-writing workshop with Latinx youth. Zamora’s visit to Charlottesville illustrates how medical departments might formally include storytelling professionals in their educational programming, and also how such initiatives can serve as opportunities for community-institutional collaboration. The sequence of these events also could be done in reverse; that is, medical education faculty could establish a collaborative partnership with an arts organization that selects and invites an artist or writer whose work is relevant to both parties.

To realize the potential of critical health humanities necessitates an expansion of the pedagogical tools used in medical education and the adaptation of a transdisciplinary approach to such education as a holistic “way of being” [8]. In transdisciplinarity, discrete disciplines do not simply exist alongside one another, but are “productively entangled” to render distinct and more nuanced understandings [5, 8]. One example of transdisciplinarity is the application of literary criticism to poetry as a teaching tool in pediatrics, as in IM’s article, “Commentary on ‘Doctor’s Office First Week in this Country,’” which explores the pedagogical use of a poem by Javier Zamora [9]. In this manuscript, IM connects literary criticism and close reading of a poem to the clinical and structural realities of caring for immigrant children.

## Strengths & challenges

Many physicians and other health care practitioners may not feel comfortable teaching critical health humanities, and we acknowledge that our unique position as experienced creative writers offers an advantage. However, we have found that our clinical experiences provide a degree of facility with guiding students to interpret the social and interpersonal conflicts portrayed in the literature and art we teach, and we have utilized film, comedy, and other genres in which we have no personal experience. Our students and colleagues at all levels also bring their own experiences and perspectives, so that our critical health humanities teaching is often an exercise in collective co-learning and meaning making, rather than didactic, one-way instruction. Furthermore, we posit that this field offers an important opportunity to create cross-campus, extramural, and/or transdisciplinary pedagogical approaches, examples of which are outlined above. Many academic medical centers are part of larger universities that include English, Art, Sociology, Anthropology, History, Philosophy, and other departments relevant to this work. Medical educators can collaborate with colleagues from these departments and with community members to develop and implement educational experiences for medical learners. Community partnerships, as outlined in the example above, can be additional sources of educational collaboration, and simultaneously exemplify the deep listening, power sharing, and collaboration that the critical health humanities aims to foster.

We recognize that many academic medical centers still lack the structures and financial resources to support sustained inter- and transdisciplinary health humanities collaboration, either within or beyond university walls. As calls increase for medical education to meaningfully address social inequities, we suggest that the creation of such collaborations may be increasingly politically tenable. We hope that the concrete examples of programming shared herein may serve as models for those hoping to establish similar educational initiatives elsewhere. In universities that have centers such as our Center for Health Humanities & Ethics, there may be opportunities to build or tap into existing interdisciplinary relationships, structures for creating or expanding courses, and protected time available for faculty. Internal or external education innovation grants may provide support for these initiatives as well. For instance, our undergraduate seminar, “Bodies & Books: Literature of Health (In)equity” was supported by a small internal grant with the explicit purpose of incentivizing innovative first-year seminars for undergraduate students.

An important challenge is the lack of time for additional content in both UME and GME curricula. Therefore, health humanities education often takes the form of elective courses, after-hours journal clubs, or optional sessions. As a result, the learners who participate in such activities are often a self-selected group. To expand the reach of this pedagogy, we recommend embedding elements of critical health humanities in preexisting educational activities, such as Grand Rounds, noon conferences, as adjuncts to existing lectures, and sessions in existing conferences and other CME content. It is crucial that institutions provide support to faculty interested in enacting these educational practices in the form of funding for faculty positions, student research, interns, administrative support, and related infrastructure for sustaining this pedagogical work [10].

## Conclusions

The critical health humanities represents an important iteration of health humanities more broadly, and it parallels increasing interest in social determinants of health and equity in health professions education. Educators can use narrative practices to teach about health equity within the frameworks of critical consciousness, structural competency, and cultural humility. In this paper we have presented several concrete examples of such educational activities, as well as strengths and challenges in the practice of critical health humanities. Future directions for this work might include the creation of new educational activities and the application of additional pedagogical frameworks. Equipping learners to appreciate the social and emotional complexities of persistent health inequities and empowering them to take action in the face of structural injustice requires creative, multi- and transdisciplinary collaborations.

## Data Availability

Not applicable.

## Abbreviations

SDoH: Social determinants of health
CHHE: Center for Health Humanities and Ethics
UME: Undergraduate medical education
GME: Graduate medical education

## References

[1] Howley L, Gaufberg E, King B (2020). The fundamental role of arts and humanities in medical education.

[2] Banaszek A (2011). Medical humanities courses becoming prerequisites in many medical schools. CMAJ.

[3] Adams Z, Reisman A (2019). Beyond sparking Joy: a call for a critical medical humanities. Acad Med.

[4] Sharma M, Pinto AD, Kumagai AK (2018). Teaching the social determinants of health: a path to equity or a road to nowhere?. Acad Med.

[5] Viney W, Callard F, Woods A (2015). Critical medical humanities: embracing entanglement, taking risks. BMJ.

[6] Metzl JM, Hansen H (2014). Structural competency: theorizing a new medical engagement with stigma and inequality. Soc Sci Med.

[7] Charon R, Hermann N, Devlin MJ (2016). Close reading and creative writing in clinical education: teaching attention, representation, and affiliation. Acad Med.

[8] Rigolot C (2020). Transdisciplinarity as a discipline and a way of being: complementarities and creative tensions. Humanit Soc Sci Commun.

[9] Mathieu IP (2022). Commentary on doctor’s office first week in this country. Acad Med.

[10] Berry S, et al. Health humanities: a baseline survey of Baccalaureate and Graduate Programs in North America. J Med Humanit. 2023;44. 10.1007/s10912-023-09790-5. 10.1007/s10912-023-09790-5 37000293

